# Japanese Encephalitis Vaccine Acceptance and Strategies for Travelers: Insights from a Scoping Review and Practitioners in Endemic Countries

**DOI:** 10.3390/vaccines11111683

**Published:** 2023-11-02

**Authors:** Punyisa Asawapaithulsert, Thundon Ngamprasertchai, Amornphat Kitro

**Affiliations:** 1 Hospital for Tropical Diseases, Faculty of Tropical Medicine, Mahidol University, Bangkok 10400, Thailand; punyisa@thaitravelclinic.com; 2 Department of Clinical Tropical Medicine, Faculty of Tropical Medicine, Mahidol University, Bangkok 10400, Thailand; thundon.ngm@mahidol.ac.th; 3 Department of Community Medicine, Chiang Mai University, Chiang Mai 50200, Thailand

**Keywords:** Japanese encephalitis, disease burden, Japanese encephalitis vaccine, vaccine acceptance, traveler

## Abstract

Japanese encephalitis (JE) remains the cause of vaccine-preventable encephalitis in individuals living in endemic areas and international travelers. Although rare, the disease’s high fatality rate emphasizes the need for effective immunization. This review aims to provide updated data on the JE burden between 2017 and 2023, vaccine acceptance, and vaccine strategies for travelers. We prospectively identified studies, using MEDLINE and PubMed, published through 2023. JE incidence has decreased in local populations and remains low among travelers from non-endemic countries. The local JE risk cannot be utilized to determine traveler risk. Adult travelers naïve to JEV infection or immunization may be at potentially higher risk. The JE vaccine acceptance rates among international travelers visiting JE endemic areas range from 0.2% to 28.5%. The cost of the vaccine and low risk perception could be barriers to JE vaccination. For travelers, an accelerated two-dose regimen of inactivated Vero cell JE vaccine (JE-VC) or a single dosage of live attenuated JE vaccine (JE-LV) may be an option. In conclusion, the JE burden among residents and travelers is lower, but the risk is not negligible. Practitioners should prioritize sharing knowledge, increasing awareness, and promoting vaccinations and preventive measures to reduce tourists’ risk of JE along their journey.

## 1. Introduction

Japanese encephalitis (JE) continues to be a vaccine-preventable encephalitis for residents of endemic areas, as well as a considerable risk to travelers from non-JE-endemic countries to endemic areas. The Japanese encephalitis virus (JEV) causes the most severe viral encephalitis, which has spread mostly in Asia (South Asia, Southeast Asia), the western Pacific, and northern Australia [1]. An estimated three billion people are living in JE endemic countries [2]. From 1998 to 2011, an estimated 67,900 JE cases occurred each year in 24 endemic countries, with an overall incidence of 1.8 per 100,000 [3]. Considering WHO case reports, published case reports, and health control statistics from endemic countries between 2017 and 2023, the incidence rate was 0.5–1 per 1,000,000 population. The estimated incidence among local residents in each country was significantly lower, with a range of up to 4668 cases in 2017 and 1886 in 2022 per year. Fatality and JE burden have gradually decreased as vaccine coverage has increased over the past decade [4,5,6,7,8,9,10,11], while 81% of cases still occurred in areas where JE vaccination programs were either in place or were being developed [3]. It is possible that some cases may be underreported.

**Figure 1:**
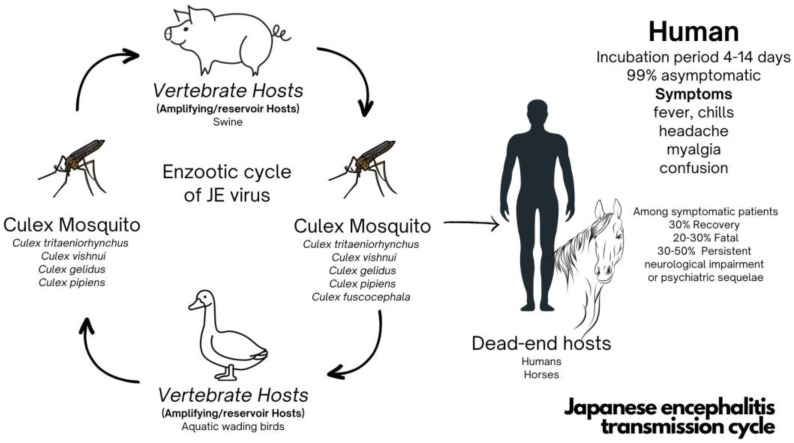
Japanese encephalitis transmission cycle.

JEV is a flavivirus that is a member of the Flaviviridae family including dengue, Zika, yellow fever, and West Nile viruses. It was isolated from *Culex tritaeniorhynchus* mosquitos for the first time in 1938. It is transmitted to humans through mosquito bites, i.e., a mosquito-borne mode, from infected Culex species, particularly *Culex tritaeniorhynchus* and other species such as *Culex vishnui*, *Culex gelidus*, and *Culex pipiens* [1,12]. The principal vertebrates, pigs and aquatic wading birds, play crucial roles as amplifying hosts. Humans are dead-end hosts; hence, there is no transmission from human to human [2,12,13]. Approximately 99% of JEV infections are asymptomatic. Following an incubation period of 4–14 days, symptomatic patients present with nonspecific prodromal symptoms, including high-grade fever, chills, headache, and myalgia, followed by neurological sequelae, including confusion, and death. Approximately 30% of symptomatic cases lead to fatality, and up to 50% of surviving patients will have neurological long-term sequelae (Figure 1). There are no specific treatments, only supportive treatments [3,14,15].

The impact of JE on domestic animals varies, leading to a range of outcomes, from asymptomatic infections in some species to acute neurological symptoms in others. In the case of horses, many instances go unnoticed as they remain asymptomatic, and the clinical disease is generally mild. Nevertheless, more severe encephalitis can occur, which can be life-threatening. Symptoms in affected horses encompass fever, lethargy, loss of appetite, and neurological signs that vary in severity. Neurological signs in horses and other animals can include coordination problems, difficulty swallowing, impaired vision, and, albeit rarely, a hyperexcitable form of the disease in adult swine. The infection does not typically manifest as symptomatic disease; however, it poses a significant reproductive challenge, resulting in issues like abortion, stillbirth, and birth defects. Infected piglets, on the other hand, can suffer from fatal neurological conditions [16].

Immunization is the most effective preventive strategy [17,18]. This review aimed to provide an overview of the JE situation between 2017 and 2023, focusing on residents and travelers. It also aimed to identify gaps in knowledge regarding JE vaccination acceptance and vaccine strategies for travelers. This study’s findings will aid healthcare practitioners in improving patient consultations, raising awareness about JE prevention, particularly among non-JE-endemic travelers planning to visit JE-endemic areas, and recommending appropriate JE vaccines based on their itinerary.

## 2. Materials and Methods

Potential studies were identified from MEDLINE via PubMed and selected studies’ reference lists through 2023. Two investigators (AK and PA) developed search strategies that were accepted by the team. If there was a discrepancy, a third reviewer (TN) would make the decision. The search terms and strategies for each database were “Japanese Encephalitis”, “traveler”, “travel”, “expatriate”, “Japanese Encephalitis vaccine”, and “acceptance”. Hand searching selected articles yielded additional references. This review only includes English articles. All relevant information was organized into four categories: an update from 2017 to 2023 for local residents and travelers, JE vaccine implications for travelers visiting JE endemic areas, vaccines under development, and time taken for the JE vaccine to become effective.

## 3. Results

The first case of JE was reported in Japan in 1871, during the largest summer–fall en-cephalitis outbreaks. In 1924, there were over 6000 incidents reported, and over 60% of them were fatal [19]. In 1934 and 1935, the JE Nakayama strain was isolated from a human case and documented beyond the region [20]. Between 1950 and 1970, JE outbreaks mostly occurred among local residents in Japan, Taiwan, and China, with morbidity rates exceeding 10 per 100,000, and sporadic cases in Vietnam, Thailand, Cambodia, Sri Lanka, Burma, and Bangladesh [18,21,22,23]. In Thailand, JE was first recorded in 1961. However, JE was not recognized as a significant public health concern until 1969, when an epidemic was reported in the Chiang Mai Valley and other areas of northern Thailand [24]. In Thailand, between 1500 and 2500 cases of JEV infections were reported annually between the 1970s and 1980s [25]. JE outbreaks were documented in Southeast Asia through 1989 [18]. In 1995, JEV emerged for the first time in northern Australia, causing an unimaginable outbreak in the Torres Strait [26]. In the Southeast Asia region, although some countries report only sporadic cases since the decades of the twentieth century, the disease is currently endemic in the geographical area [27]. However, reported cases of JEV infection decreased dramatically after JE vaccine implementation in national immunization programs (NIP). Since the 1990s, significant progress has been achieved in JE surveillance and the implementation of immunization programs, including an increase in JE vaccine coverage from 9% in 2009 to 82% in 2021 in India and from 87.4% in 2006 to 95.08% in 2019 in Thailand [28].

### 3.1. A Comprehensive Update from 2017 to 2023 for Local Residents and Travelers

WHO case reports, published case reports, and endemic country health control statistics from 2017 to 2023 revealed that there has been a decrease in the incidence of JE among both residents and travelers, but the majority of countries where the JEV is still endemic are popular travel destinations, including India, China, Vietnam, Myanmar, Malaysia, the Philippines, Thailand, and Indonesia [4].

**Figure 2:**
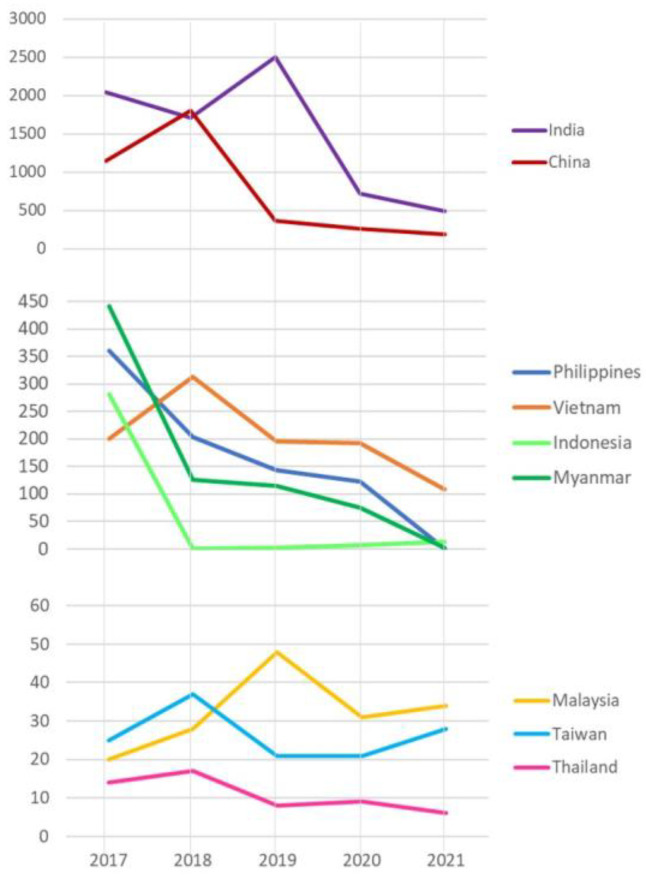
Japanese encephalitis among local populations residing in JE-endemic countries, 2017–2021 (This figure illustrates only the officially confirmed cases of JEV infection, with some milder cases likely going unreported due to either a lack of testing or individuals not seeking testing).

Interestingly, India had an observably high rate of JEV infection among local residents between 2017 and 2021, with almost 2500 cases per year in 2019, followed by China with roughly 1800 cases per year in 2018. Between 2017 and 2018, the Philippines and Vietnam both recorded a significant number of cases, up to 361 and 313 cases, respectively [4]. Moreover, the number of cases in Thailand is estimated to be less than 20 per year, with fourteen, seventeen, eight, nine, and three cases each year from 2017 to 2022, respectively [8,9,10]. The overall JE incidence trend in Thailand, the Philippines, China, India, and Vietnam steadily declined between 2017 and 2022 (Figure 2).

**Figure 3:**
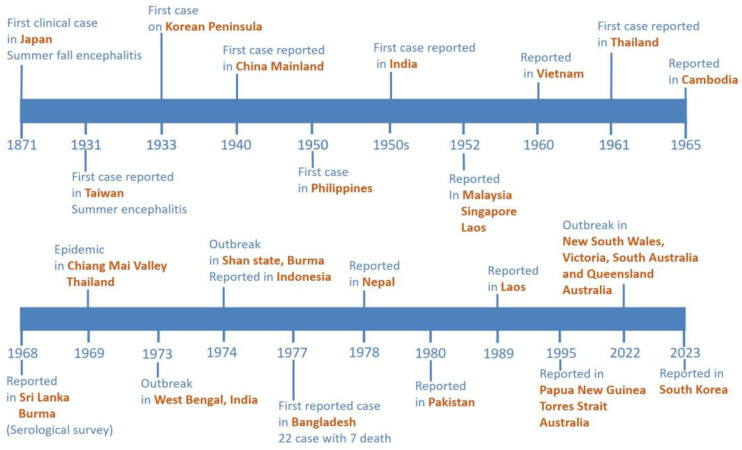
Epidemic timeline of Japanese encephalitis.

Taiwan and Malaysia both reported a comparable number of JE-infected individuals. However, the Philippines, Taiwan, Indonesia, and Malaysia have fewer than 40 cases every year [4,5,6]. The latest WHO report covers data up to 2022 but includes reports from only eight Southeast Asian countries: 108 cases from Vietnam, 99 cases from the Philippines, 24 cases from Malaysia, nine cases from Lao PDR, six cases from Myanmar, two cases from Cambodia, one case from Timor-Leste, and 0 cases from Thailand. India reported the highest number of JEV infection cases, with 1271, followed by 137 cases in China, 82 cases in Bangladesh, and 79 cases in Nepal [4]. In March 2022, Queensland officials identified human JE in four Australian states (Queensland, Victoria, New South Wales, and South Australia). Three mortalities were reported [26,29]. Last updated on 6 September 2023, the Korea Disease Control and Prevention Agency confirmed the first suspected case of Japanese encephalitis in the Korean population. The affected individual is a 60-year-old male with no vaccination record [30] (Figure 3).

The reduction of new JEV infections among residents can be attributed to various factors, such as effective JE surveillance, the successful implementation of NIPs, and changes in lifestyle patterns. Additionally, the transformation of rural regions into peri-urban areas and the encroachment of pig farms into residential zones also contribute to this decline [17,31].

**Figure 4:**
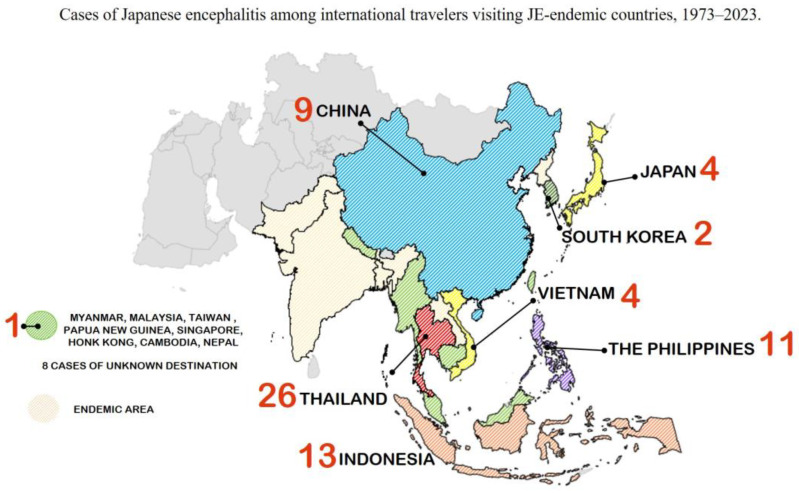
Reported Japanese encephalitis cases among international travelers visiting endemic countries, 1973–2023.

No new cases have been documented among non-JE-endemic travelers who contracted a JEV infection at their destinations between 2017 and 2023 (Figure 4). Over the past 30 years, the approximate annual incidence rate among American travelers was less than one in one million [17,32]. Furthermore, the estimated risk of JEV infection within one month for rural tourists during transmission season could be in the range of 1 in 5000–200,000 population per week [17,31,32]. Regardless of type or duration of stay, the risk estimation for JEV infection among Swedish and Finnish travelers to Thailand was 1: 257,000–400,000 travelers [33,34]. Only 85 cases of travel-associated JE in travelers who had visited endemic regions were identified in 15 countries between 1973–2023, and none of them had been immunized against JE [32,34,35]. Twenty-six cases were reported among international travelers in Thailand, followed by Indonesia with 13, the Philippines with 11, China with nine, Vietnam with four, Japan with four, and South Korea with two cases. Additionally, one case per country was recorded from Myanmar, Malaysia, Taiwan, Singapore, Papua New Guinea, Hong Kong, Cambodia, and Nepal [32,34,35,36].

### 3.2. Japanese Encephalitis Vaccine Implications for Travelers Visiting JE-Endemic Areas

Multiple vaccines against the JEV have been developed and studied since the 1940s. Vaccines can be classified into two categories including inactivated and live attenuated JE vaccines (Table 1). There was limited information available about the clinical use of the JE vaccine among international travelers. The majority of JE vaccine acceptance trials are conducted in pre-travel consultation clinics among westerners such as Americans, British nationals, Europeans, and Australians [37,38,39,40]. Varying levels of acceptance were targeted among Western travelers. Among those planning to visit JE-endemic countries, Australian travelers exhibited the highest acceptance rate at 28.5%, followed by Americans (4–28%), and Swiss/British (0.2–0.3%). The proportion of Australian travelers who were JE-vaccinated was the highest among exchange student programs/school placement (51.1%), followed by business travelers (43.2%), visitors to rural areas (34.6%), and short-term travelers (22.4%) [41]. Those American travelers who received JE vaccines mostly had plans to travel for 30 days or more during the JE transmission season or to visit rural areas in SEA [37]. Among those who did not receive the JE vaccine, their reasons included lack of time, cost, lack of awareness, and a perception of low risk of contracting the illness [37,38,39,40].

#### 3.2.1. Inactivated JE Vaccine

The earliest vaccination against JEV was a formalin-inactivated virus generated from mouse brains (MB) and manufactured in Japan. The Nakayama-NIH strain of the JEV was first used in humans in 1954, and it was eventually replaced by the Beijing-1 strain in 1988 [42]. The efficacy of first-generation immunization ranges from 91 to 97.5% and lowers the incidence of JE illnesses in Japan, Thailand, and Taiwan [43,44,45].

The mouse brain JE (MB-JEV) vaccine available to travelers from Europe and the Western Hemisphere was named JE-VAX, which had been licensed in the United States in 1992 and was discontinued in 2005 due to serious side effects, including acute disseminated encephalomyelitis (ADEM) and other potentially life-threatening facial and upper airway angioedema and severe hypersensitivity, occurring at a rate of 1 to 17 per 10,000 vaccine recipients. Due to a rare occurrence of ADEM in a vaccinated person, the manufacture of the MB-JEV vaccine was removed and production discontinued in 2005, and the last remaining stockpiles expired in May 2011 [30,42,46].

Next-generation inactivated JEV vaccines, containing the Beijing-3 or P-3 strains of the JEV grown in primary hamster kidney cells and manufactured in China, have been used routinely in all Chinese national vaccine campaigns since 1968 and branded as JEBIK, ENCEVAC, and JEVAC^TM^ [42,47,48,49]. Three doses of an inactivated cell culture JE vaccine administered at days 0, 7, and 28 at 12–24 months of age resulted in a 100% seroprotection rate after second and third doses among recipients [46,47]. This vaccine was exclusively produced in China and served as the primary JE vaccination until the year 2000. It was removed from the vaccine program due to the availability and accessibility of the live attenuated SA 14-14-2 vaccine [50]. The last JE vaccine containing the Beijing-3 or P-3 strains was the freeze-dried vaccine branded as JEVACTM, which has been registered in China since 2008. A three-shot series regimen was administered, with the first shot on day 0, the second shot 1–4 weeks later, and a booster vaccination at one year. The seroprotection rates at one year after the primary series and one month after the booster were 89.3% and 100%, respectively [48].

A JE vaccine grown in Vero cells (JE-VC) derived from the attenuated SA-14-14-2 was introduced in January 2009, branded under IXIARO^®^ in the United States, JESPECT^®^ in Australia and New Zealand, and JEEV^®^ in India [49]. It was licensed for children in the U.S. as young as two months old in 2013 [32]. JE-VCs administered in an accelerated two-dose regimen within one week generated a significant immune response comparable to the standard 28-day schedule (two doses administered 28 days apart) seroconversion rate, up to 99–100% in both groups at 28 days after the second JE-VC. At 10–12 months after the second JE-VC, the seroprotection was 94% for the accelerated group and 88% for the conventional group [50,51]. Following the completion of the full 28-day schedule, the seroprotection rates were measured at different time intervals. At one month, the rate was 96%, followed by 83% at six months, 58% at 12 months, and 48% at 24 months. One study suggested that antibodies could persist for approximately 82% of the 60-month duration after the initial dose of the conventional 28-day series [50,52]. However, seroprotection in persons over the age of 65 who completed a two-dose primary series decreased gradually after six weeks [53].

When the individuals only received a single dose of JE-VC, seroconversion was around 41% and 26% on day 28 and day 56 after the first dosage, respectively [54]. A booster dose of JE-VC given 15 months after a two-dose conventional series demonstrated protective neutralizing antibodies in up to 100% of participants at 28 days and in about 96% of individuals 76 months after the booster dose [54,55].

JENVAC^®^ is produced and licensed in India since 2014. It utilizes the JEV Kolar-821564XY strain, which was isolated from a clinical case of JEV infection in an endemic region of India and grown in Vero cells. A two-dose regimen revealed a seroprotection rate of over 90% after a 28-day interval between doses. A seroprotection rate persisted at over 60% for two years of follow-up [56]. A single dose of JENVAC^®^ revealed the seroprotection rate reached 92.4% after four weeks and above 80% after a two-year follow-up [57].

#### 3.2.2. Live Attenuated JE Vaccine

Only two newly introduced live Japanese encephalitis vaccines (JE-LV) are available on the market. One is called CD. JEVAX^®^, which consists of an attenuated SA 14-14-2 virus grown in hamster kidney cell culture. The effectiveness of a single dose of JE-LV provided to children was 94.5% at six months and 96.2% at five years [58,59]. The other vaccine is called IMOJEV^®^, which is a recombinant chimeric virus vaccine that combines the yellow fever vaccine 17D-204 (CV-JE) with JE components and was introduced in 2012. Neutralizing antibodies were detected in 99% of vaccinated individuals 28 days after vaccination. In addition, the seroprotection rate was 93%, persisting for up to five years [60]. The analysis study suggests that a single dose of JE-CV confers to most adults a high level of protection against JE for at least ten years [61]. It is currently authorized in Australia, Thailand, Malaysia, the Philippines, Hong Kong, and Singapore [49].

### 3.3. Vaccines under Development

According to the UNICEF Supply Division, one JE vaccine is in phase 3 of human studies, developed by the Chinese Academy of Medical Sciences in China. Furthermore, there was a relatively low level of activity in the development of innovative pipeline products related to JE vaccines [62].

Recent research uncovered only three JE vaccine candidates. Firstly, there are virus-like particles (VLPs), created in vitro, which include the envelope structural protein of JEV expressed through *Pichia pastoris*. This VLP vaccine triggered a strong immune response, both humeral and cellular immunity, in both mouse and pig models. It provided full protection against a lethal JEV challenge in immunodeficient mice. Moreover, when pigs were immunized with the VLP alone, without an adjuvant, they produced elevated levels of neutralizing antibodies against JEV [63].

Secondly, a JEV variant that was unable to replicate due to a deletion in the non-structural-1 (NS1) region, known as JEV-ΔNS1, demonstrated notable safety, exceptional genetic stability, and a significant reduction in its ability related to neuroinvasiveness and neurovirulence in mice. After a single dose, it exhibited strong immunogenicity in mice and offered a degree of protection comparable to that of the SA14-14-2 vaccine [64].

Thirdly, an inactivated vaccine, utilizing the highly virulent JEV P3 strain and containing numerous crucial immunogenic epitopes, was subcloned into the pVAX1 vector (pV) to create pV-JP3ME, a DNA vaccine aimed at enhancing its immunogenicity. This resulted in the detection of elevated levels of IgG antibodies and neutralizing antibodies against JEV in mice [65].

All of the new designs for the JEV subunit vaccine using the VLP technique, the NS1-deleted JE vaccine, and the subcloned DNA vaccine have shown promise as potential JEV vaccine candidates against JEV.

### 3.4. Time Taken for the JE Vaccine to Become Effective

The effectiveness of the JE vaccine depends on the specific vaccine used and the dosing schedule. As for JE vaccines grown in Vero cells (JE-VC), such as IXIARO, nearly 60% of individuals showed seroconversion within 10 days after the initial dose, and 100% achieved seroconversion just 7 days after the second immunization. As for the live SA14-14-2 JE vaccine, research conducted by Bista et al. and Tandan et al. [59,66] in Nepal showed an efficacy exceeding 98% (95% CI: 94.9–100%) within a median period of two weeks after vaccination. As for the JE chimeric virus vaccine (JE-CV), when administered according to the regular schedule with doses given on day 0 and day 28, only 15.4% of JE-CV vaccine-naïve children demonstrated seroprotection after seven days. However, after the administration of a booster dose of JE-CV, 96.2% achieved seroprotection within seven days. After 28 days from the JE-CV booster dose, 100% of children achieved seroprotection [67].

It is important to note that even after the initial dose(s), the vaccine may not provide immediate protection. It would take about 1 to 2 weeks after the final dose for the immune system to build sufficient protection against JE [42].

**Table 1:** Summary of Japanese Encephalitis Vaccine.

Vaccine Type	Substrate	Trade Name	Manufacturer	Vaccine Strains	License	Regimen	Efficacy Study	Prices (In USD)	Country
Inactivated MB-JEV	Mouse brain	BIKEN^®^, JE-VAX, Sanofi Pasteur	BIKEN, Osaka, Japan (Research Foundation for Microbial Diseases of Osaka University)	Nakayama strain Beijing-1 strain	Japan in 1954	Days 0, 7, 28 at 12–24 months of age; booster after 12 months, then every 3–5 years	In Thailand, 84.8–100% [45] 85.59% after 1 dose [43] 91.07% after 2 doses [43] 98.51% after 3 doses [43]	-	European Union, United States, India, Japan, Malaysia, North Korea, South Korea, Sri Lanka, Taiwan, Thailand, Vietnam
Inactivated	Hamster kidney cells	-	China	Beijing-3 or P-3	China in 1968	-	-	-	China
Inactivated (Freeze dried)	Vero cells	JEBIK^®^	BIKEN, Japan (JEBIK-V)	Beijing-1	Japan in 2009	Days 0, 7, 28 at 12–24 months of age; booster after 12 months, then every 3–5 years	-		Japan
	Vero cells	ENCEVAC^®^	KAKETSUKEN, Kumamoto, Japan (ENCEVAC, JEIMMUGEN)	Beijing-1	Japan in 2011	Days 0, 7, 28 at 12–24 months of age; booster after 12 months, then every 3–5 years	SPRs 100% after 2 doses and 3 doses [47]		Japan and South Korea
	Vero cells	JEVAC^TM^	Liaoning Cheng Da Biotechnology Co., Ltd., Shenyang, China	Beijing P-3 strain	China in 2008	Day 0, 1–4 weeks and a booster vaccination at one year	SPRs 83% after 2 doses and SPRs 100% after booster [48]	-	China
Inactivated JE-VC	Vero cells	IXIARO^®^ (USA, EU); JESPECT^®^ (AUS, NZ); JEEV^®^	Valneva Scotland Ltd., Livingston, UK; Biological E, Telangana, India	SA-14-14-2	USA, Australia, and Europe in 2009	Days 0, 28 as early as 2 months of age; booster after 1 year; accelerated schedule: Days 0,7	SPRs 86–98% [51,53]	96–339 [68,69]	European Union, United States, Canada, Latin America, Australia, New Zealand, Japan, Hong Kong, South Korea, Singapore, India, Nepal, Bangladesh, Bhutan, Pacific Islands, Papua New Guinea
Inactivated	Vero cells	JENVAC^®^	Bharat Biotech International Ltd., Telangana, India	Kolar-821564XY	India in 2014	Days 0, 28 as early as 6 months of age	SPRs 61.7–99.8% [56,57]	8–15 [70]	India
Live attenuated	Hamster kidney cells	CD.JEVAX^®^	Chengdu Institute of Biological Products (CDIBD), Chengdu, China	SA-14-14-2	China in 1988	A single dose at the age of 8 month and older; if needed, booster at 3–12 month	78–99.3% after single dose [42,58,66,71,72]	13.84–36.6 [73]	Japan, South Korea, China, Hong Kong, India, Nepal, Sri Lanka, Thailand
Chimera	Vero cells	IMOJEV^®^	Government Pharmaceutical Organization-Merieux Biological Products Co., Chachoengsao, Thailand; Sanofi Pasteur, Val-de-Reuil, France	JE SA-14-14-2/Yellow fever 17 D	Australia and Thailand in 2012	A single dose at the age of 9 months and older; booster after 12 to 24 months	SPRs 87–99% [60,61]	13.32–43 [73]	Australia, South Korea, Thailand

## 4. Discussion

This is the initial review conducted on the JE situation between 2017–2023, along with its implications for travelers. The incidence of JEV infection in the local endemic population has declined, and the number of cases among travelers has remained relatively low with low acceptance rates towards JE vaccination, approximately 0.2% to 28.5% among international travelers visiting Southeast Asia [39,40].

Despite a downward trend in reported cases of JE and reduced risk, it remains a significant concern due to high morbidity and mortality, especially for older adult travelers naïve to JEV infection or immunization who may be at a higher risk of progression to encephalitis, as this trend had been noted in Australia and Korea, previously non-endemic countries. The risk of JE in the local population cannot be used to directly estimate the risk of infection among travelers, as the former receive NIPs immunization. This could lead to an epidemiological silence, which may falsely create a perception of low risk for travelers visiting SEA, and some cases of international travelers may not be published or may be underreported [74]. The implementation of JE vaccines in NIPs has resulted in a decrease in infection incidence among local populations [11,75]. However, with insufficient vaccine coverage, outbreaks can still occur, as observed in India and China [45,76]. Healthcare providers play a crucial role in raising awareness and providing adequate information about JE. They should prioritize recommending JE vaccination to residents residing in endemic areas, particularly those born before the availability of JE vaccines. It is essential to advise travelers, including those planning short-term trips but engaging in high-risk activities during dusk and dawn in rural or peri-urban areas, particularly during the rainy season from May to October. However, travelers at non-peak times should also prioritize vaccination for a safe trip.

The cost of the JE vaccine was a key factor contributing to the low uptake of JE vac-cination among travelers. This trend was confirmed by a study conducted among Aus-tralian travelers visiting Bali, Indonesia, that found that the expense of JE vaccination in their home country acted as a barrier to getting vaccinated [77]. To illustrate, the price of a single dose of IMOJEV^®^ in Thailand is 13 USD, whereas it is 65 USD in Bali, which is significantly more affordable than the cost of 195 USD for a single dose in Australia. Additionally, this cost comparison is even more favorable when compared to the two-dose regimen of IXIARO^®^, which comes to a total of 540 USD for two doses [68,69,70,73,77]. Promoting JE vaccination for budget-conscious travelers and large families planning group trips who cannot afford the vaccine in their home country but can obtain it at their destination could boost vaccine acceptance rates.

Several JE vaccines are available on the market with varying prices, schedules, and side effects. For last-minute travelers, an accelerated schedule of IXIARO^®^ administered seven days apart can offer protection up to 88% two years after vaccination [50,51]. Western travelers can choose a single dose of CD.JEVAX^®^ or IMOJEV^®^, which are licensed, cheaper, and available in Thailand and many Southeast Asian countries. Those are effective and provide long-lasting immunity for up to five years after a single vaccination [40,60,61]. It is advisable to continue practicing mosquito avoidance measures even after two weeks following JE vaccination to reduce the risk of contracting other mosquito-borne diseases like malaria, dengue, and chikungunya. Implementing this strategy could result in enhanced protection for travelers visiting JE-endemic areas, particularly those who are last-minute or budget travelers. Consequently, using CD.JEVAX^®^ or IMOJEV^®^ as a booster dose for individuals who have previously received JE-VC may be a promising strategy to enhance their immunity.

This study has several limitations. The majority of the data was collected from Western countries, which reflect only Western perspectives. The lower utilization of diagnostic tests, the existence of asymptomatic cases, and the reliance on published cases in this review may lead to a potential underestimation of the true disease burden among both local populations and travelers. Hence, it is essential to incorporate the perspective of practitioners residing in JE-endemic countries. This could contribute to the improvement of vaccination rates and the delivery of better pre-travel consultations. Additionally, it would be interesting to conduct future studies on seroprotection among heterologous vaccine platforms by priming with inactivated vaccines and boosting with live attenuated vaccines.

## 5. Conclusions

The JE burden among residents and travelers has decreased, but the risk is not negligible. Furthermore, acceptance of the JE vaccine among international travelers remains low due to low-risk perceptions and the high price of the vaccine in their home country. Practitioners should prioritize sharing knowledge, increasing awareness, and promoting vaccinations and preventive measures to minimize the risk of infection, especially among those elderly and naïve to JEV infection or immunization. For travelers who are unable to complete the vaccine before departing their homelands, an accelerated JE Vero cell (JE-VC) or a single dose of live vaccination (JE-LV) may be an alternative.

## Data Availability

Data sharing not applicable.
